# Patient‐reported outcomes in older breast cancer survivors with and without prior chemotherapy treatment

**DOI:** 10.1002/cam4.6394

**Published:** 2023-08-08

**Authors:** Taiwo Adesoye, Kai‐Ping Liao, Susan Peterson, Liang Li, Daria Zorzi, Holly M. Holmes, Mariana Chavez‐MacGregor, Sharon H. Giordano

**Affiliations:** ^1^ Department of Breast Surgical Oncology The University of Texas MD Anderson Cancer Center Houston Texas USA; ^2^ Department of Health Services Research The University of Texas MD Anderson Cancer Center Houston Texas USA; ^3^ Department of Behavioral Science, Division of Cancer Prevention and Population Sciences The University of Texas MD Anderson Cancer Center Houston Texas USA; ^4^ Department of Biostatistics The University of Texas MD Anderson Cancer Center Houston Texas USA; ^5^ Division of Geriatric and Palliative Medicine, Department of Internal Medicine University of Texas Houston McGovern Medical School Houston Texas USA; ^6^ Department of Breast Medical Oncology The University of Texas MD Anderson Cancer Center Houston Texas USA

**Keywords:** breast cancer, geriatric oncology, survivorship

## Abstract

**Background:**

Little is known about long‐term treatment‐related symptoms in older breast cancer survivors. We characterized long‐term patient‐reported symptoms and examined factors associated with the presence and severity of symptoms, and symptom interference with daily activities.

**Methods:**

Texas Cancer Registry (TCR) Medicare linkage data was used to identify breast cancer patients age 65 and older with local/regional stage disease diagnosed between 2012–2013. Symptom burden was assessed using breast‐specific items from the Patient‐Reported Outcomes version of the Common Terminology Criteria for Adverse Events (PRO‐CTCAE™). Demographic and clinical data also were collected. Logistic regression models were used to assess the association between symptom burden and respondent sociodemographic and clinical characteristics.

**Results:**

Of 4448 eligible patients, 1594 (response‐rate 35.8%) completed questionnaires. Of these, 1245 eligible respondents were included in the analysis based on self‐reported data. Median time from diagnosis to survey completion was 68 months (IQR: 62–73). Most frequently reported symptoms were fatigue/lack of energy (76.8%), aching muscles (72.1%) and aching joints (72.5%). Receipt of chemotherapy was associated with higher symptom burden. Patients treated with adjuvant chemotherapy had higher risk of numbness/tingling (OR: 3.16; 95% CI: 2.36–4.24), hair loss (OR: 2.72; 95% CI: 2.05–3.60), and fatigue/lack of energy (OR: 1.80; 95% CI: 1.29–2.52). Similarly, patients who received chemotherapy were more likely to report the majority of symptoms as moderate to severe and as interfering with daily activities.

**Conclusion:**

Receipt of chemotherapy is associated with significant symptom burden more than 5 years after breast cancer treatment. Long‐term chemotherapy impact should be discussed with patients in a shared‐decision making process and approaches to symptom management during survivorship care are needed.

## 1 INTRODUCTION

The number of breast cancer survivors in the United States is estimated at 3.8 million and over 60% are 65 years and older.^1^, ^2^, ^3^ Although chemotherapy has contributed to improved survival outcomes,^4^, ^5^ both short‐term and late effects of treatment are common.^6^ Little is known about patient‐reported symptoms and potential side effects of treatment experienced by breast cancer survivors years after their primary treatment, particularly among older women, who are vulnerable to treatment‐related toxicities.

Traditionally, in clinical trials, assessments of symptomatic adverse effects have focused on safety using provider‐collected Common Terminology Criteria for Adverse Events (CTCAE).^7^ Importantly, patient‐reported outcomes (PROs) provide insight regarding a patient's perspective of treatment effects and more accurately capture the impact of therapy on quality‐of‐life.^8^, ^9^, ^10^ Health‐related quality‐of‐life (HRQOL) measures have been developed and utilized in routine cancer care, but they have a limited focus on therapeutic toxicity.^11^, ^12^, ^13^ To better capture toxicity profiles of therapies directly from patients, the National Cancer Institute’ (NCI) developed the Patient‐Reported Outcomes version of the Common Terminology Criteria for Adverse Events (PRO‐CTCAE™) as a companion to the CTCAE.^14^, ^15^, ^16^

The objective of this study was to characterize long‐term patient‐reported adverse effects using items from the PRO‐CTCAE™. In a population‐based cohort of older breast cancer survivors, we investigated factors associated with the presence of symptoms, severity of symptoms, symptom interference with daily activities, and the impact of prior chemotherapy on such symptoms.

## 2 METHODS

### 2.1 Study population

This study was approved by both the Institutional Review Boards of MD Anderson and the Texas Department of State Health Services. Eligible cancer survivors were identified from the Texas Cancer Registry (TCR) and Medicare‐linked data, and included patients who were age 65 or older, were diagnosed with local/regional breast cancer between 2012–2013, and were Medicare beneficiaries with Part A and B coverage and without HMO enrollment for 12 months continuously after their diagnosis. TCR provided names and mailing addresses for 4726 Texas residents, and the physician of record. Of these, 278 were identified as deceased or as having undeliverable addresses, and the survey was mailed to 4448 patients who met eligibility criteria.

### 2.2 Study measures and data collection

This study was one of the research projects of the Comparative Effectiveness Research on Cancer in Texas (CERCIT) program, funded by the Cancer Prevention and Research Institute of Texas (CPRIT). Patient‐reported outcomes, demographic and clinical variables were collected using a self‐administered questionnaire, data from TCR, and Medicare claims. The primary outcome measure was derived from the PRO‐CTCAE™, a PRO measurement system to evaluate symptomatic toxicity over a 7‐day recall period in patients receiving cancer treatment.^16^ The study questionnaire included items assessing nine treatment‐related adverse effects were selected from the PRO‐CTCAE™ item library, including; arm/leg swelling, hair loss, numbness/tingling, problem with concentration, problem with memory, aching muscles, aching joints, fatigue/lack of energy, and hot flashes. Frequency (never/rarely/occasionally/frequently/almost constantly), severity (none/mild/moderate/severe/very severe) and interference with daily activities (not at all/a little bit/ somewhat/quite a bit/very much) were assessed for each adverse effect. Three weeks prior to contacting eligible patients, the physician of record received mailed notification of the study, per TCR requirements. Eligible patients received a mailed study invitation letter in English and Spanish with a study questionnaire in English and compensation worth 10 dollars.^17^, ^18^ Reminder letters were sent to non‐respondents at 2, 4–6, and 8–10 weeks after the initial mailing. Data were collected between April 2018 and October 2019.

Age at diagnosis, marital status, disease stage and hormone receptor status were obtained from TCR data. Race/ethnicity, education, household income, height and weight were self‐reported. Charlson co‐morbidity score was constructed using Klabunde's algorithm,^17^ using inpatient and outpatient claims within a 12‐month window preceding the 30‐day timeframe of diagnosis. Receipt of radiation therapy and type of surgery were determined based on Current Procedural Terminology (CPT) codes and ICD‐9 procedure codes abstracted from MEDPAR, NCH and OUTPAT claim files in the 9‐month treatment window post‐diagnosis. Receipt of endocrine therapy was determined using national drug codes (NDC) from Medicare Part‐D claims in 12 months post the diagnosis date in addition to self‐reported use. Receipt of chemotherapy and chemotherapeutic agents was identified using Healthcare Common Procedure Coding System (HCPCS) J codes within 6 months after the diagnosis date.

### 2.3 Statistical analysis

To evaluate differences between respondents and non‐responders, inverse probability weighing (IPW) was applied to the analysis to address potential non‐response bias. For each case, we computed the probability of being selected from a pool of 4099 eligible cases. The inversed value of the probability was used as a weight of each observation to balance out the bias due to non‐response. Finally, the normalized inversed probability was generated by dividing the inverse probability by the mean and used as the final weight in the analysis.^19^ Rao‐Scott Chi‐Square was used to examine the association of chemotherapy with respondent sociodemographic and clinical variables. Similarly, we examined presence, severity, and interference of self‐reported symptoms between individuals receiving chemotherapy or not. Logistic regression models were conducted to examine the association of symptom presence, symptom severity, and symptom interference with respondent sociodemographic and clinical variables. We included odds ratios (OR) and 95% CIs to present the likelihood of symptom presence, symptom severity, and symptom interference. We conducted a stepwise selection regression analysis for covariate selection and included the following variables in our initial model; age at diagnosis, gender, marital status, BMI, race/ethnicity, education, household income, Charlson comorbidity, receipt of chemotherapy, radiation therapy, breast/axillary surgery, and tumor stage.^20^ Variables that met the significance threshold of *p* < 0.05 were included in the final models. Data analyses were performed using SAS (version 9.4 SAS Institute Inc.).

## 3 RESULTS

**FIGURE 1:**
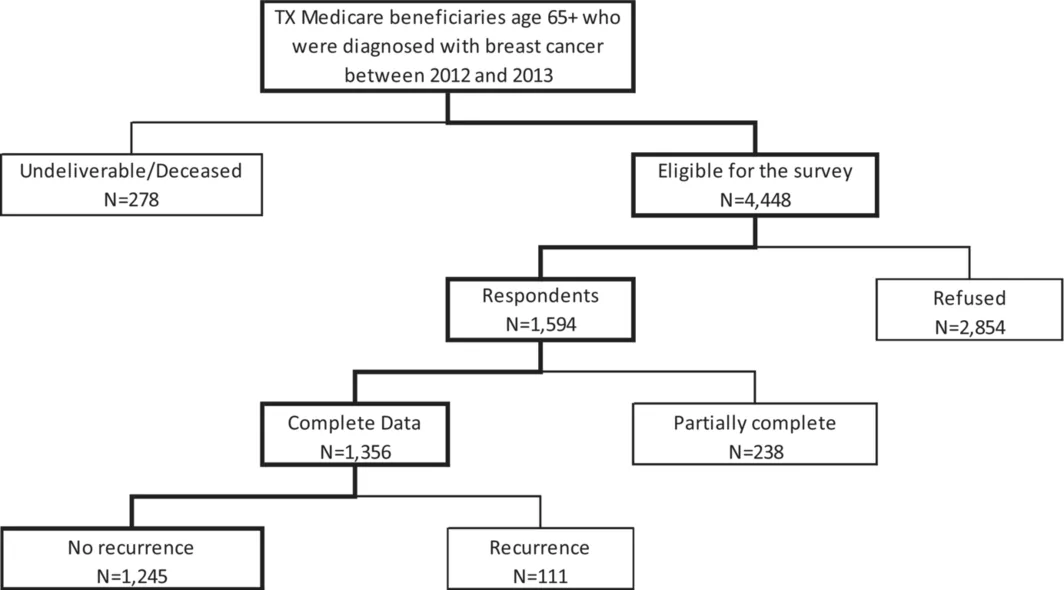
Selection of study participants.

Of 4448 eligible patients who were mailed a study invitation and questionnaire, 2854 declined or did not respond, and 1356 evaluable questionnaires were completed. Due to self‐reported recurrent disease, 111 questionnaires were excluded from analysis (Figure 1). Compared to non‐respondents, respondents were younger, more likely be married, White, with no comorbidities, and had received chemotherapy (Table S1). Median survey return time was 37 days (IQR: 21–70 days).

A total of 1245 eligible respondents were included in the analyses. Median time from diagnosis to survey completion was 68 months (IQR: 62–73). Demographic and clinical characteristics of these patients are summarized in Table 1. Overall, 98.9% (*n* = 1231) of respondents were female. Median age at diagnosis was 71 years (IQR: 67–75), and median age at survey completion was 76 years (IQR: 73–81). The majority of respondents were white, overweight or obese and without comorbidities. Hormone receptor positive disease was present in 76.4% (*n* = 951) of patients and 77.2% (*n* = 961) of patients had localized disease. Lumpectomy was performed in 57.4% (*n* = 715) of patients, and 37.4% (*n* = 465) received mastectomy.

**TABLE 1:** Demographic, clinical and treatment characteristics of survey respondents by receipt of chemotherapy.

	Total		Chemotherapy				Rao‐Scott *p*‐value
	*N* = 1245		Yes (*N* = 366)		No (*N* = 879)		
	*N*	Weighted %	*N*	Weighted %	*N*	Weighted %	
Age at diagnosis^a^							
65–69	533	36.3	191	36.1	342	63.9	<0.0001
70–74	381	26.2	114	28.9	267	71.1	
75–79	200	20.2	43	20.9	157	79.1	
80+	131	17.4	18	11.2	113	88.8	
Marital status^a^							
Married	507	37.3	143	26.7	364	73.3	0.9878
Not married	264	27.5	85	26.6	179	73.4	
UNK	474	35.2	138	27.1	336	72.9	
Race and ethnicity (self‐reported)^c^							
White non‐Hispanic/other^d^	1061	82.5	295	25.4	766	74.6	0.0242
Black	72	6.6	33	41.4	39	58.6	
Hispanic	112	10.9	38	28.7	74	71.3	
BMI							
<25	390	30.9	111	26.5	279	73.5	0.0143
25–30	399	31.6	107	24.4	292	75.6	
>30	326	26.0	117	33.4	209	66.6	
Missing	130	11.6	31	19.9	99	80.1	
Education^c^							
High school or under/missing^d^	445	38.5	138	28.7	307	71.3	0.3426
Some college or 2 years degree	403	31.7	123	27.5	280	72.5	
College graduate	196	15.5	49	21.8	147	78.2	
More than a 4 years college degree	201	14.4	56	25.9	145	74.1	
Income^c^							
Less than $19,999	139	12.8	44	28.6	95	71.4	0.1211
$20,000–$49,999	356	28.6	111	28.4	245	71.6	
$50,000–$99,999	299	22.3	94	29.7	205	70.3	
$100,000 or more	180	12.9	54	28.2	126	71.8	
UNK/Mis	271	23.4	63	20.5	208	79.5	
Charlson Comorbidity Score^b^							
0	784	56.3	229	27.2	555	72.8	0.7247
1	269	22.7	86	28.2	183	71.8	
2+	153	17.9	39	23.7	114	76.3	
UNK	39	3.1	12	29.4	27	70.6	
Diagnosis year^a^							
2012	609	47.1	170	26.2	439	73.8	0.6705
2013	636	52.9	196	27.4	440	72.6	
Stage at diagnosis^a^							
Localized	961	75.7	202	19.1	759	80.9	<0.0001
Regional	284	24.3	164	50.9	120	49.1	
Hormone receptor positive (ER+/PR+)^a^							
Yes	951	78.4	226	21.9	725	78.1	<0.0001
No	294	21.6	140	44.8	154	55.2	
Radiation therapy^b^							
Yes	747	52.3	214	26.8	533	73.2	0.9999
No	498	47.7	152	26.8	346	73.2	
Endocrine^b^							
Yes	596	47.0	144	22.5	452	77.5	0.0019
No	649	53.0	222	30.7	427	69.3	
Doxorubicin							
Yes	125	8.8	125	100.0	0	0.0	N/A
No	1120	91.2	241	19.8	879	80.2	

*Note*: Other category for race includes Native American, Asian, Pacific Islander.

Abbreviations: ER, endocrine receptor; PR, progesterone receptor.

^a^ Data obtained from Texas Cancer Registry.

^b^ Data obtained from Medicare claims.

^c^ Data is self‐reported.

^d^ Categories are combined for confidentiality to avoid any cell size *N* < 11.

Chemotherapy was administered to 29.4% (*n* = 366) of patients and of these, 34.2% (*n* = 125) received doxorubicin, while 88.5% (*n* = 324) received a taxane (Table S2). Of patients who received chemotherapy, 55.2% (*n* = 202) had localized disease. A total of 50.8% (*n* = 632) of patients received endocrine therapy and of these patients, 16% (*n* = 101) reported current use.

Compared to patients who did not receive chemotherapy, patients who received chemotherapy were younger, white and normal weight and were more likely to have been diagnosed with regional stage and hormone receptor negative disease (Table 1).

### 3.1 Patient‐reported outcomes

#### 3.1.1 Presence of symptoms

Overall, 93.5% (*n* = 1162) of patients reported at least 1 symptom in the prior 7 days. The most commonly reported symptoms were fatigue/lack of energy (76.8%), aching muscles (72.1%) and aching joints (72.5%). These symptoms were also more likely to have higher mean score on severity and interference with daily activities (Table S3).

**FIGURE 2:**
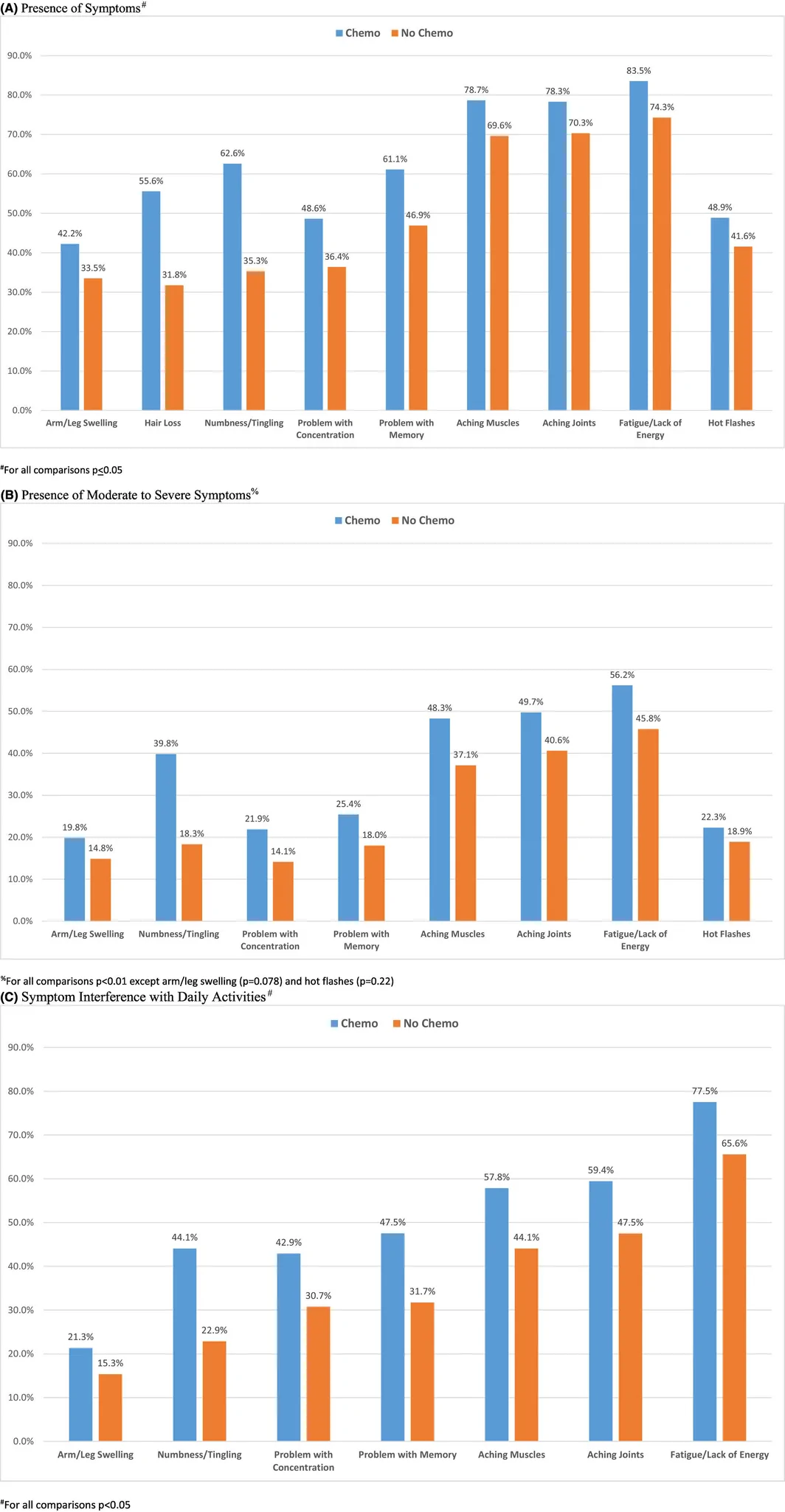
Patient‐reported symptoms by receipt of chemotherapy. (A) Presence of symptoms^#^. ^#^For all comparisons *p* ≤ 0.05. (B) Presence of moderate to severe symptoms^%^. ^%^For all comparisons *p* < 0.01 except arm/leg swelling (*p* = 0.078) and hot flashes (*p* = 0.22). (C) Symptom interference with daily activities^#^. ^#^For all comparisons *p* < 0.05.

Patients who received chemotherapy were more likely to report experiencing all symptoms compared to those who did not receive chemotherapy (Figure 2A) Compared to those who did not receive chemotherapy, those who did reported differences in numbness/tingling (62.6% vs. 35.3%, *p* < 0.0001), hair loss (55.6% vs. 31.8%, *p* < 0.0001), and problems with memory (61.1% vs. 46.9%, *p* < 0.0001).

**FIGURE 3:**
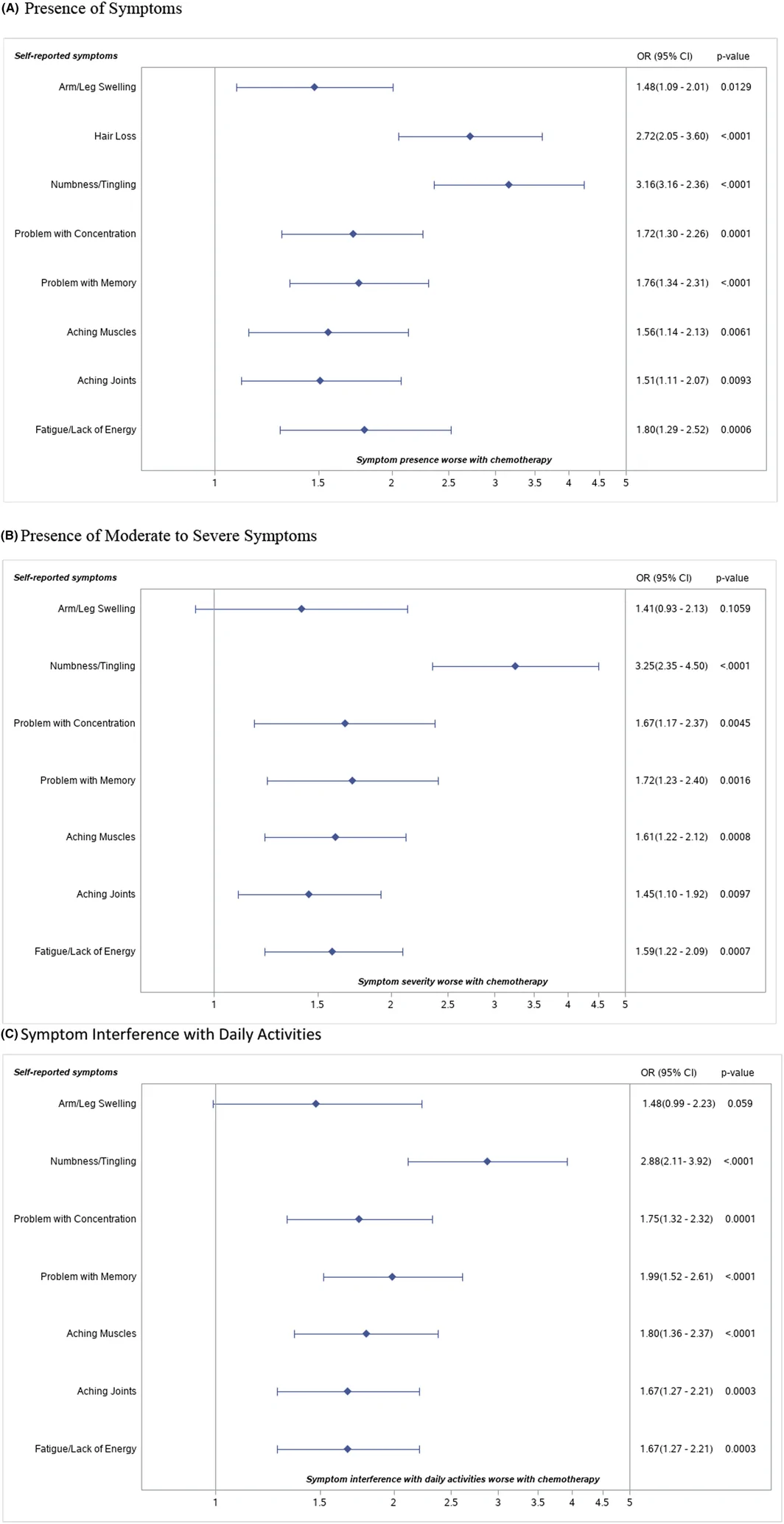
Logistic regression models of breast cancer patient reported symptoms and association with receipt of chemotherapy. (A) Presence of symptoms. (B) Presence of moderate to severe symptoms. (C) Symptom interference with daily activities.

In logistic regression models to examine the association of symptom presence with respondent sociodemographic and clinical variables, receipt of chemotherapy was independently associated with the presence of all symptoms except hot flashes (Figure 3A). The highest estimates of effect size were noted for numbness/tingling (OR = 3.16; 95% CI: 2.36–4.24), hair loss (OR = 2.72; 95% CI: 2.05–3.60), and fatigue/lack of energy (OR = 1.80; 95% CI: 1.29–2.52) (Figure 3A, Table S4A). BMI was also a significant predictor of symptoms and patients who were overweight or obese were more likely to report the presence of all symptoms compared to normal weight patients except for hot flashes (Table S4A). Similarly, compared to patients with no comorbidities, patients with one or more comorbidities were more likely to report the presence of all symptoms except for aching muscles and hot flashes (Table S4A). Having a lower income was associated with the presence of extremity swelling compared to patients who had localized disease. Patients with lower income were more likely to report extremity swelling, numbness/tingling, problems with concentration, problems with memory, aching muscles, and aching joints. Compared to white patients, black patients were more likely to report numbness/tingling (OR = 2.63; 95% CI: 1.46–4.74) and hot flashes (OR = 2.20; 95% CI: 1.25–3.88) (Table S4A).

#### 3.1.2 Symptom severity

Overall, 71% (*n* = 869) of patients reported at least one symptom as moderate to severe in the preceding 7 days. The most severe symptoms included fatigue/lack of energy (48.6%), aching joints (43.1%) and aching muscles (40.1%) (Table S3). Compared to patients who did not receive chemotherapy, a higher proportion of patients who received chemotherapy reported moderate to severe symptoms for all symptoms except hot flashes (Figure 2B). The highest differences were noted for numbness/tingling (39.8% vs. 18.3%, *p* < 0.0001), aching muscles (48.3% vs. 37.1%, *p* = 0.001) and fatigue/lack of energy (56.2% vs. 45.8%, *p* = 0.002).

In logistic regression models, patients who received chemotherapy were more likely to report all symptoms as moderate to severe except hot flashes (Figure 3B). Similarly, the presence of one or more comorbidities independently predicted the presence of moderate to severe symptoms (Table S4B). Compared to patients with localized disease, patients with regional disease were more likely to report severe extremity swelling (OR = 19.4; 95% CI: 1.27–2.95). Overweight and obese BMI were also strongly associated with majority of symptoms and, compared to normal weight, obese patients were more likely to report extremity swelling (OR = 5.41; 95% CI: 3.08–9.53), numbness/tingling (OR = 1.62; 95% CI: 1.05–2.48), fatigue/lack of energy (OR = 1.60; 95% CI: 1.14–2.24) and aching joints (OR = 2.63; 95% CI: 1.88–3.69) (Table S4B).

#### 3.1.3 Symptom interference with daily activities

A total of 77.1% (*n* = 950) patients reported that at least one symptom interfered with their daily activities. Fatigue/lack of energy (68.8%), aching joints (50.7%) and aching muscles (47.8%) were the symptoms most reported as interfering with daily activities (Table S3). Patients who received chemotherapy were significantly more likely to report interference of daily activities by all symptoms (Figure 2B). The largest differences were seen for numbness/tingling (44.1% vs. 22.9%, *p* < 0.0001), aching muscles (57.8% vs. 44.1%, *p* < 0.0001) and problems with memory (47.5% vs. 31.7%, *p* < 0.0001). Receipt of doxorubicin was associated with symptom interference of daily activity compared to receipt of other chemotherapy regimens or no chemotherapy (Table S3).

In logistic regression models assessing interference of symptoms with daily activities, receipt of chemotherapy, higher number of comorbidities and overweight or obese BMI were independently associated with all symptoms evaluated (Figure 3C, Table S4C).

## 4 DISCUSSION

To our knowledge, this is the first study evaluating patient‐reported long‐term adverse effects in a population‐based sample of older breast cancer survivors and how these symptoms differ by prior receipt of chemotherapy. Using the PRO‐CTCAE™ questionnaire administered approximately 5–6 years after diagnosis, we found that patients who previously received chemotherapy were more likely to report the presence of symptoms, a higher severity of symptoms and symptom interference of their daily activities. In adjusted analyses, receipt of chemotherapy was strongly associated with greater symptom burden. These findings suggest that receipt of chemotherapy confers a higher risk of adverse effects that persists well beyond treatment and into survivorship.

Chemotherapy‐related toxicities among breast cancer patients have been well documented during therapy and in the short‐term post‐treatment, particularly in clinical trials.^4^, ^6^, ^21^, ^22^, ^23^, ^24^, ^25^ In our study, 93.3% of patients reported at least 1 symptom in the prior 7 days and 69.8% reported at least one symptom as moderate to severe. Patients who received doxorubicin were more likely to report symptoms compared to patients who received other chemotherapy regimens or no chemotherapy. Receipt of chemotherapy was the strongest predictor of physical and cognitive symptoms with regards to presence, severity or interference with daily activity. Our results expand upon studies describing toxicities with receipt of chemotherapy within a year of diagnosis.^26^, ^27^, ^28^, ^29^ The iCanCare population‐based study examined frequency and severity of treatment‐related toxicities using a scaled patient‐reported measure (completed, on average, 7 months after surgery) and found that receipt of chemotherapy was associated with higher toxicity severity.^30^ Using the EORTC QLQ C30, Bottomely et al demonstrated that chemotherapy negatively impacts HRQOL in a dose related fashion in the first few months of therapy.^31^

In this study, median time from diagnosis to survey completion and symptom reporting was 68 months suggesting that treatment‐related symptoms persist well beyond active treatment. Late chemotherapy treatment effects such as cardiotoxicity,^32^, ^33^ premature ovarian failure,^34^ and development of future cancers^35^ have been described, but there are limited data on patient experience after therapy, especially in older patients who are less likely to participate in clinical trials and more likely to experience toxicities related to chemotherapy. Earlier studies suggested that decline in HRQOL secondary to chemotherapy may be transient.^21^, ^31^, ^36^, ^37^ Ganz et al evaluated QOL in breast cancer survivors 3 years after various adjuvant treatments and found no significant differences in energy/fatigue, emotional functioning or overall QOL using standardized measures of HRQOL including the Medical Outcomes Study Short Form‐36 (MOS‐SF‐36).^37^ However, after 6 years of follow‐up, survivors treated with chemotherapy had lower global QOL compared to survivors who did not receive systemic therapy.^38^ In our study, it is unclear whether symptoms persist after receipt of chemotherapy or if they develop years after therapy as PRO measures were not collected at multiple time points. Furthermore, in the elderly population, some symptoms may be secondary to normative aging. Nonetheless, these findings of greater symptoms with receipt of chemotherapy have important implications for informed decision making regarding chemotherapy use among older breast cancer patients^39^, ^40^ Additionally, it is critical to ensure the development of an effective long‐term symptom management plan as a priority for survivorship care.^41^, ^42^, ^43^

In our comorbidity assessment, patients with one or more co‐morbidities within 12 months of cancer diagnosis were more likely to report that physical symptoms (extremity swelling, numbness/tingling, aching muscles, fatigue/lack of energy) and cognitive symptoms (problems with concentration and problems with memory) interfered with their daily activities. A meta‐analysis by Edwards et al evaluating the influence of comorbidities on adjuvant chemotherapy use for early breast cancer found that while patients with comorbidities received less chemotherapy, they experienced greater toxicity compared to their counterparts with no comorbidities.^44^ However, other studies have demonstrated that despite higher grade toxicities in older patients compared to younger patients, treatment is still well tolerated and majority will complete therapy.^45^ Interestingly, decline in HRQOL in breast cancer survivors has been shown to vary by age.^46^, ^47^ When compared to an age‐matched general population, younger patients experienced a larger decline in QOL in the immediate post‐treatment period with subsequent improvement while older patients showed a gradual decrease over time.^47^ Cancer management in this population is challenging as forgoing curative intent therapy to avoid treatment‐related toxicity may impact cancer‐related survival. Validated comprehensive geriatric assessment (CGA) tools and toxicity prediction tools such as the Chemotherapy Risk Assessment Scale for High‐Age Patients (CRASH) and Cancer and Aging Research Group's (CARG) may help guide treatment decisions for older patients.^48^, ^49^, ^50^, ^51^, ^52^

We observed a strong association between higher BMI at the time of survey completion and reported symptom incidence and severity. Previous studies found a higher severity of chemotherapy‐related peripheral neuropathy (CIPN) in patients with a higher baseline BMI.^29^, ^53^ A cross‐sectional study evaluated the impact of obesity on CIPN and overall symptom burden in cancer survivors and found that patients with coexisting obesity and CIPN reported higher symptom severity and pain using a patient‐reported questionnaire.^54^ In this study, it is unknown whether respondents who reported high BMI on the study questionnaire also had elevated BMI prior to treatment. Regardless, we observed an association of current BMI with symptom incidence and severity. This is hypothesis‐generating as our data suggests that high BMI in survivorship increases risk for symptom burden potentially independent of BMI prior to treatment. Our findings also support increased surveillance for symptom burden and mitigation among patients with high BMI prior to treatment and among patients who develop obesity in survivorship.^55^

Study limitations include its observational design using claims data as unmeasured variables may account for some of our findings. Utilizing Medicare claims to accurately ascertain receipt of chemotherapy also has limitations which varies by disease site and chemotherapy type.^56^ We implemented standard methodology for minimizing non‐response in population‐based surveys; however, our response rate was 35.8%.^57^, ^58^ This may limit generalizability of our findings given differences between responders and non‐responders as described. The PRO‐CTCAE™ measure was included as part of a larger study questionnaire that conferred a potentially greater respondent burden, which also may have contributed to the response rate. While we utilized the validated PRO‐CTCAE™ measure to elicit chemotherapy toxicity, this measure was not explicitly designed to assess long‐term toxicities and additional toxicities pertinent to geriatric populations may be uncaptured. Additionally, we did not administer the survey at multiple time points which subjects our findings to recall bias and limits our ability to understand patterns of symptom development over time. We also have no information on symptom management through the follow up period, utilization of healthcare services, and subsequent impact on severity.

## 5 CONCLUSION

In summary, our findings demonstrate significant symptom burden associated with chemotherapy more than 5 years after breast cancer diagnosis. This data highlights the importance of patient‐centered discussion of each patient's risk of adverse events and benefit from chemotherapy in a shared‐decision making approach. Accurate and timely diagnosis of symptoms during and after cancer treatment is essential to optimize symptom management and improve quality of life for cancer survivors. Our study findings highlight the need for such assessment and support long after active treatment.

## AUTHOR CONTRIBUTIONS

**Taiwo Adesoye:** Data curation (supporting); visualization (supporting); writing – original draft (lead); writing – review and editing (lead). **Kai‐Ping Liao:** Data curation (lead); formal analysis (lead); methodology (supporting); writing – review and editing (supporting). **Susan Peterson:** Conceptualization (supporting); data curation (lead); investigation (lead); methodology (lead); writing – review and editing (supporting). **Liang Li:** Conceptualization (supporting); formal analysis (lead); methodology (lead); writing – review and editing (supporting). **Daria Zorzi:** Investigation (supporting); project administration (lead); writing – review and editing (supporting). **Holly M. Holmes:** Conceptualization (supporting); writing – review and editing (supporting). **Mariana Chavez‐MacGregor:** Conceptualization (supporting); writing – review and editing (supporting). **Sharon H. Giordano:** Conceptualization (lead); funding acquisition (lead); investigation (lead); methodology (lead); writing – original draft (supporting); writing – review and editing (supporting).

## FUNDING INFORMATION

This study was supported by the Cancer Prevention and Research Institute of Texas (CPRIT) grant RP160674, and in part by the Susan G. Komen Breast Cancer Foundation grant SAC150061, SAC22022, BCRF‐22‐190, and by the National Institutes of Health/National Cancer Institute through Cancer Center Support grant P30CA016672.

## CONFLICT OF INTEREST STATEMENT

None.

## Supporting information


Table S1‐S4.

## Data Availability

Data from this manuscript will be made available for submission.
