# Supplementary material for: Patient‐reported outcomes in older breast cancer survivors with and without prior chemotherapy treatment
Source: Cancer Med. 2023 Aug 8;12(17):17740–52. doi: 10.1002/cam4.6394 (PMC10524015; doi:10.1002/cam4.6394)
Supplement: Supplementary file 1 — Table S1‐S4. [file CAM4-12-17740-s001.docx]

**Supplemental Tables and figures**

**eTable 1.** Demographic, Clinical and treatment Characteristics of Survey Respondents and Non-respondents (online only)

|  | **Total** | | **Responders** | | **Non-responders** | |  |
| --- | --- | --- | --- | --- | --- | --- | --- |
|  | **(N=4099)** | | **(N=1245)** | | **(N=2854)** | | **p-value** |
|  | N | % | N | % | N | % |  |
| Age at diagnosis* |  |  |  |  |  |  | <.0001 |
| 65-69 | 1402 | 34.2 | 533 | 42.8 | 869 | 30.5 |  |
| 70-74 | 1140 | 27.8 | 381 | 30.6 | 759 | 26.6 |  |
| 75-79 | 796 | 19.4 | 200 | 16.1 | 596 | 20.9 |  |
| 80+ | 761 | 18.6 | 131 | 10.5 | 630 | 22.1 |  |
| Gender |  |  |  |  |  |  | 0.451 |
| Male | 39 | 1.0 | 14 | 1.1 | 25 | 0.9 |  |
| Female | 4060 | 99.1 | 1231 | 98.9 | 2829 | 99.1 |  |
| Marital status* |  |  |  |  |  |  | <.0001 |
| Married | 1506 | 36.7 | 507 | 40.7 | 999 | 35.0 |  |
| Not married | 1118 | 27.3 | 264 | 21.2 | 854 | 29.9 |  |
| UNK | 1475 | 36.0 | 474 | 38.1 | 1001 | 35.1 |  |
| Race/Ethnicity * |  |  |  |  |  |  | <.0001 |
| White non-Hispanic | 3251 | 79.3 | 1048 | 84.2 | 2203 | 77.2 |  |
| Black | 270 | 6.6 | 67 | 5.4 | 203 | 7.1 |  |
| Hispanic | 476 | 11.6 | 110 | 8.8 | 366 | 12.8 |  |
| Others | 102 | 2.5 | 20 | 1.6 | 82 | 2.9 |  |
| Diagnosis year* |  |  |  |  |  |  | 0.4692 |
| 2012 | 1970 | 48.1 | 609 | 48.9 | 1361 | 47.7 |  |
| 2013 | 2129 | 51.9 | 636 | 51.1 | 1493 | 52.3 |  |
| Charlson Comorbidity Score^#^ |  |  |  |  |  |  | <.0001 |
| 0 | 2313 | 56.4 | 784 | 63.0 | 1529 | 53.6 |  |
| 1 | 929 | 22.7 | 269 | 21.6 | 660 | 23.1 |  |
| 2+ | 725 | 17.7 | 153 | 12.3 | 572 | 20.0 |  |
| UNK | 132 | 3.2 | 39 | 3.1 | 93 | 3.3 |  |
| Stage at Diagnosis* |  |  |  |  |  |  | 0.1425 |
| Localized | 3103 | 75.7 | 961 | 77.2 | 2142 | 75.1 |  |
| Regional | 996 | 24.3 | 284 | 22.8 | 712 | 25.0 |  |
| Hormone receptor positive* (ER+/PR+) |  |  |  |  |  |  | 0.4178 |
| Yes | 3164 | 77.2 | 951 | 76.4 | 2213 | 77.5 |  |
| No | 935 | 22.8 | 294 | 23.6 | 641 | 22.5 |  |
| Chemotherapy^#^ |  |  |  |  |  |  | 0.0018 |
| Yes | 1072 | 26.2 | 366 | 29.4 | 706 | 24.7 |  |
| No | 3027 | 73.9 | 879 | 70.6 | 2148 | 75.3 |  |
| Surgery^#^ |  |  |  |  |  |  | 0.0003 |
| Lumpectomy | 2164 | 52.8 | 715 | 57.4 | 1449 | 50.8 |  |
| Mastectomy | 1679 | 41.0 | 465 | 37.4 | 1214 | 42.5 |  |
| None/UNK | 256 | 6.3 | 65 | 5.2 | 191 | 6.7 |  |
| Radiation therapy^#^ |  |  |  |  |  |  | <.0001 |
| Yes | 2133 | 52.0 | 747 | 60.0 | 1386 | 48.6 |  |
| No | 1966 | 48.0 | 498 | 40.0 | 1468 | 51.4 |  |

^*^Data obtained from Texas Cancer Registry

^#^Data obtained from Medicare claims

Abbreviations: *ER endocrine receptor, PR progesterone receptor*

Other category for race includes Native American, Asian, Pacific Islander

**eTable 2**. Chemotherapy treatment in Older Women with Breast Cancer (N=366)(online only)

| **Chemotherapy agent** | **N** | **%** | **Cumulative N** | **Cumulative %** |
| --- | --- | --- | --- | --- |
| Adriamycin + Paclitaxel or Docetaxel | 111 | 30.33 | 111 | 30.33 |
| Adriamycin without Paclitaxel or Docetaxel | 14 | 3.83 | 125 | 34.15 |
| Paclitaxel or Docetaxel without Adriamycin | 213 | 58.2 | 338 | 92.35 |
| Others | 28 | 7.65 | 366 | 100 |

**eTable 3**. Distribution of Symptoms by Presence, Severity and Interference with Daily Activities in Older Women with Breast Cancer Measured by the PRO-CTCAE (N=1,245) (online only)

|  | Presence in prior 7 days | | Moderate-severe severity | | Interference with daily activity | |
| --- | --- | --- | --- | --- | --- | --- |
| **Symptom** | N | Weighted % | N | Weighted % | N | Weighted % |
| Arm/Leg Swelling | 412 | 35.8 | 172 | 16.2 | 185 | 16.9 |
| Hair Loss | 471 | 38.1 | - | N/A | - | N/A |
| Numbness/Tingling | 514 | 42.6 | 279 | 24.1 | 335 | 28.5 |
| Problem with Concentration | 481 | 39.7 | 196 | 16.2 | 402 | 34.0 |
| Problem with Memory | 630 | 50.7 | 229 | 20.0 | 445 | 36.0 |
| Aching Muscles | 884 | 72.1 | 471 | 40.1 | 559 | 47.8 |
| Aching Joints | 879 | 72.5 | 510 | 43.1 | 607 | 50.7 |
| Fatigue/Lack of Energy | 938 | 76.8 | 563 | 48.6 | 837 | 68.8 |
| Hot Flashes | 569 | 43.5 | 261 | 19.8 | - | N/A |

**eTable 4**

A: Logistic Regression Models of Long-term Symptoms Reported by Breast Cancer Survivors – Symptom Presence

|  | **Arm/Leg Swelling^#^** | | | **Hair Loss** | | | **Numbness/Tingling** | | | **Problem with Concentration** | | | **Problem with Memory** | | |
| --- | --- | --- | --- | --- | --- | --- | --- | --- | --- | --- | --- | --- | --- | --- | --- |
|  | **OR** | **95% CI** | **p-value** | **OR** | **95% CI** | **p-value** | **OR** | **95% CI** | **p-value** | **OR** | **95% CI** | **p-value** | **OR** | **95% CI** | **p-value** |
| **Age at Dx** |  |  |  | Not Included | | | Not Included | | | Not Included | | | Not Included | | |
| **65-69** | 1.00 |  |  |  |  |  |  |  |  |  |  |  |  |  |  |
| **70-74** | 1.10 | (0.80-1.51) | 0.544 |  |  |  |  |  |  |  |  |  |  |  |  |
| **75-79** | 1.01 | (0.68-1.48) | 0.976 |  |  |  |  |  |  |  |  |  |  |  |  |
| **80+** | 1.93 | (1.20-3.12) | 0.007 |  |  |  |  |  |  |  |  |  |  |  |  |
| **Race and Ethnicity (Self-reported)** | Not Included | | |  |  |  |  |  |  | Not Included | | | Not Included | | |
| **White non-Hispanic** |  |  |  | 1.00 |  |  | 1.00 |  |  |  |  |  |  |  |  |
| **Black** |  |  |  | 1.34 | (0.78-2.31) | 0.293 | 2.63 | (1.46-4.74) | 0.001 |  |  |  |  |  |  |
| **Hispanic** |  |  |  | 2.09 | (1.33-3.28) | 0.002 | 1.29 | (0.80-2.08) | 0.291 |  |  |  |  |  |  |
| **Others** |  |  |  | 1.08 | (0.48-2.45) | 0.854 | 0.38 | (0.16-0.92) | 0.032 |  |  |  |  |  |  |
| **BMI** |  |  |  |  |  |  |  |  |  |  |  |  |  |  |  |
| **<25** | 1.00 |  |  | 1.00 |  |  | 1.00 |  |  | 1.00 |  |  |  |  |  |
| **25-30** | 1.72 | (1.17-2.52) | 0.005 | 1.15 | (0.82-1.60) | 0.428 | 1.20 | (0.85-1.70) | 0.301 | 1.89 | (1.35-2.64) | 0.000 |  |  |  |
| **>30** | 3.34 | (2.29-4.89) | <.0001 | 1.75 | (1.25-2.47) | 0.001 | 1.94 | (1.35-2.78) | 0.000 | 1.69 | (1.20-2.39) | 0.003 |  |  |  |
| **Missing** | 2.36 | (1.46-3.82) | 0.001 | 2.36 | (1.45-3.82) | 0.001 | 2.34 | (1.42-3.85) | 0.001 | 1.71 | (1.05-2.79) | 0.033 |  |  |  |
| **Marital status** | Not Included | | | Not Included | | |  |  |  |  |  |  | Not Included | | |
| **Married** |  |  |  |  |  |  | 1.00 |  |  | 1.00 |  |  |  |  |  |
| **Not married** |  |  |  |  |  |  | 0.65 | (0.45-0.95) | 0.025 | 0.87 | (0.61-1.23) | 0.423 |  |  |  |
| **UNK** |  |  |  |  |  |  | 0.61 | (0.45-0.83) | 0.002 | 0.73 | (0.55-0.97) | 0.032 |  |  |  |
| **Income** | Not Included | | | Not Included | | |  |  |  | Not Included | | |  |  |  |
| **Less than $19,999** |  |  |  |  |  |  | 1.00 |  |  |  |  |  | 1.00 |  |  |
| **$20,000-$49,999** |  |  |  |  |  |  | 1.02 | (0.64-1.61) | 0.943 |  |  |  | 0.98 | (0.63-1.51) | 0.917 |
| **$50,000-$99,999** |  |  |  |  |  |  | 0.90 | (0.55-1.46) | 0.656 |  |  |  | 0.98 | (0.63-1.54) | 0.936 |
| **$100,000 or more** |  |  |  |  |  |  | 0.58 | (0.33-1.01) | 0.053 |  |  |  | 0.72 | (0.43-1.22) | 0.223 |
| **UNK/Mis** |  |  |  |  |  |  | 0.61 | (0.37-1.01) | 0.053 |  |  |  | 0.56 | (0.35-0.90) | 0.017 |
| **Charlson** |  |  |  | Not Included | | |  |  |  |  |  |  |  |  |  |
| **0** | 1.00 |  |  |  |  |  | 1.00 |  |  | 1.00 |  |  | 1.00 |  |  |
| **1** | 1.35 | (0.97-1.88) | 0.078 |  |  |  | 1.74 | (1.25-2.41) | 0.001 | 1.26 | (0.93-1.72) | 0.140 | 1.24 | (0.91-1.68) | 0.169 |
| **2+** | 2.35 | (1.58-3.50) | <.0001 |  |  |  | 2.53 | (1.67-3.82) | <.0001 | 1.76 | (1.18-2.63) | 0.006 | 1.70 | (1.14-2.53) | 0.009 |
| **UNK** | 1.27 | (0.63-2.55) | 0.499 |  |  |  | 2.02 | (0.97-4.20) | 0.060 | 0.68 | (0.33-1.42) | 0.306 | 0.61 | (0.30-1.26) | 0.180 |
| **Chemotherapy** |  |  |  |  |  |  |  |  |  |  |  |  |  |  |  |
| **No** | 1.00 |  |  | 1.00 |  |  | 1.00 |  |  | 1.00 |  |  | 1.00 |  |  |
| **Yes** | 1.48 | (1.09-2.01) | 0.013 | 2.72 | (2.05-3.60) | <.0001 | 3.16 | (2.36-4.24) | <.0001 | 1.72 | (1.30-2.26) | 0.000 | 1.76 | (1.34-2.31) | <.0001 |
| **Dx Stage** |  |  |  | Not Included | | | Not Included | | | Not Included | | | Not Included | | |
| **Localized** | 1.00 |  |  |  |  |  |  |  |  |  |  |  |  |  |  |
| **Regional** | 1.45 | (1.05-2.01) | 0.026 |  |  |  |  |  |  |  |  |  |  |  |  |
|  |  |  |  |  |  |  |  |  |  |  |  |  |  |  |  |
|  | **Aching Muscles** | | | **Aching Joints** | | | **Fatigue/Lack of Energy** | | | **Hot Flashes** | | |  |  |  |
|  | **OR** | **95% CI** | **p-value** | **OR** | **95% CI** | **p-value** | **OR** | **95% CI** | **p-value** | **OR** | **95% CI** | **p-value** |  |  |  |
| **Age at Dx** | Not Included | | | Not Included | | | Not Included | | |  |  |  |  |  |  |
| **65-69** |  |  |  |  |  |  |  |  |  | 1.00 |  |  |  |  |  |
| **70-74** |  |  |  |  |  |  |  |  |  | 0.66 | (0.50-0.87) | 0.003 |  |  |  |
| **75-79** |  |  |  |  |  |  |  |  |  | 0.47 | (0.33-0.67) | <.0001 |  |  |  |
| **80+** |  |  |  |  |  |  |  |  |  | 0.25 | (0.15-0.42) | <.0001 |  |  |  |
| **Race and Ethnicity (Self-reported)$** | Not Included | | | Not Included | | | Not Included | | |  |  |  |  |  |  |
| **White non-Hispanic** |  |  |  |  |  |  |  |  |  | 1.00 |  |  |  |  |  |
| **Black** |  |  |  |  |  |  |  |  |  | 2.20 | (1.25-3.88) | 0.006 |  |  |  |
| **Hispanic** |  |  |  |  |  |  |  |  |  | 1.37 | (0.81-2.33) | 0.236 |  |  |  |
| **Others** |  |  |  |  |  |  |  |  |  | 0.76 | (0.36-1.64) | 0.490 |  |  |  |
| **BMI** |  |  |  |  |  |  |  |  |  | Not Included | | |  |  |  |
| **<25** | 1.00 |  |  | 1.00 |  |  | 1.00 |  |  |  |  |  |  |  |  |
| **25-30** | 1.44 | (1.03-2.01) | 0.033 | 1.94 | (1.39-2.72) | 0.000 | 1.62 | (1.15-2.29) | 0.006 |  |  |  |  |  |  |
| **>30** | 2.23 | (1.53-3.24) | <.0001 | 2.70 | (1.86-3.91) | <.0001 | 1.75 | (1.19-2.57) | 0.005 |  |  |  |  |  |  |
| **Missing** | 1.75 | (1.05-2.91) | 0.032 | 1.99 | (1.22-3.25) | 0.006 | 1.40 | (0.82-2.38) | 0.212 |  |  |  |  |  |  |
| **Marital status** | Not Included | | |  |  |  |  |  |  | Not Included | | |  |  |  |
| **Married** |  |  |  | 1.00 |  |  | 1.00 |  |  |  |  |  |  |  |  |
| **Not married** |  |  |  | 1.05 | (0.71-1.57) | 0.797 | 1.06 | (0.71-1.57) | 0.780 |  |  |  |  |  |  |
| **UNK** |  |  |  | 0.68 | (0.50-0.92) | 0.012 | 0.70 | (0.51-0.96) | 0.026 |  |  |  |  |  |  |
| **Income** |  |  |  |  |  |  | Not Included | | | Not Included | | |  |  |  |
| **Less than $19,999** | 1.00 |  |  | 1.00 |  |  |  |  |  |  |  |  |  |  |  |
| **$20,000-$49,999** | 0.51 | (0.30-0.87) | 0.013 | 0.48 | (0.28-0.83) | 0.008 |  |  |  |  |  |  |  |  |  |
| **$50,000-$99,999** | 0.72 | (0.42-1.23) | 0.228 | 0.49 | (0.28-0.84) | 0.010 |  |  |  |  |  |  |  |  |  |
| **$100,000 or more** | 0.44 | (0.25-0.79) | 0.006 | 0.33 | (0.18-0.61) | 0.000 |  |  |  |  |  |  |  |  |  |
| **UNK/Mis** | 0.53 | (0.31-0.91) | 0.022 | 0.40 | (0.23-0.69) | 0.001 |  |  |  |  |  |  |  |  |  |
| **Charlson** | Not Included | | |  |  |  |  |  |  | Not Included | | |  |  |  |
| **0** |  |  |  | 1.00 |  |  | 1.00 |  |  |  |  |  |  |  |  |
| **1** |  |  |  | 1.09 | (0.78-1.52) | 0.625 | 1.53 | (1.06-2.21) | 0.025 |  |  |  |  |  |  |
| **2+** |  |  |  | 2.49 | (1.48-4.20) | 0.001 | 2.71 | (1.57-4.67) | 0.000 |  |  |  |  |  |  |
| **UNK** |  |  |  | 0.72 | (0.36-1.47) | 0.369 | 0.97 | (0.43-2.17) | 0.937 |  |  |  |  |  |  |
| **Chemotherapy** |  |  |  |  |  |  |  |  |  | Not Included | | |  |  |  |
| **No** | 1.00 |  |  | 1.00 |  |  | 1.00 |  |  |  |  |  |  |  |  |
| **Yes** | 1.56 | (1.14-2.13) | 0.006 | 1.51 | (1.11-2.07) | 0.009 | 1.80 | (1.29-2.52) | 0.001 |  |  |  |  |  |  |

^#^Gender is omitted

B: Logistic Regression Models of Long-term Symptoms Reported by Breast Cancer Survivors – Symptom Severity

|  | **Arm/Leg Swelling** | | | **Numbness/Tingling** | | | **Problem with Concentration** | | | **Problem with Memory** | | |
| --- | --- | --- | --- | --- | --- | --- | --- | --- | --- | --- | --- | --- |
|  | **OR** | **95% CI** | **p-value** | **OR** | **95% CI** | **p-value** | **OR** | **95% CI** | **p-value** | **OR** | **95% CI** | **p-value** |
| **Age at diagnosis** |  |  |  | Not Included | | | Not Included | | |  |  |  |
| **65-69** | 1.00 |  |  |  |  |  |  |  |  | 1.00 |  |  |
| **70-74** | 0.76 | (0.48-1.21) | 0.253 |  |  |  |  |  |  | 1.00 | (0.69-1.45) | 0.998 |
| **75-79** | 1.85 | (1.15-2.99) | 0.012 |  |  |  |  |  |  | 1.31 | (0.83-2.06) | 0.247 |
| **80+** | 2.32 | (1.19-4.52) | 0.013 |  |  |  |  |  |  | 2.14 | (1.25-3.66) | 0.006 |
| **Race and Ethnicity (Self-reported)** | Not Included | | | Not Included | | |  |  |  | Not Included | | |
| **White non-Hispanic** |  |  |  |  |  |  | 1.00 |  |  |  |  |  |
| **Black** |  |  |  |  |  |  | 1.25 | (0.67-2.35) | 0.484 |  |  |  |
| **Hispanic** |  |  |  |  |  |  | 0.41 | (0.20-0.86) | 0.018 |  |  |  |
| **Others** |  |  |  |  |  |  | 2.82 | (0.70-11.40) | 0.145 |  |  |  |
| **BMI** |  |  |  |  |  |  | Not Included | | | Not Included | | |
| **<25** | 1.00 |  |  | 1.00 |  |  |  |  |  |  |  |  |
| **25-30** | 2.50 | (1.33-4.71) | 0.005 | 1.11 | (0.72-1.69) | 0.641 |  |  |  |  |  |  |
| **>30** | 5.41 | (3.08-9.53) | <.0001 | 1.62 | (1.05-2.48) | 0.028 |  |  |  |  |  |  |
| **Missing** | 2.86 | (1.36-6.00) | 0.006 | 2.64 | (1.50-4.65) | 0.001 |  |  |  |  |  |  |
| **Marital status** | Not Included | | |  |  |  | Not Included | | | Not Included | | |
| **Married** |  |  |  | 1.00 |  |  |  |  |  |  |  |  |
| **Not married** |  |  |  | 0.64 | (0.41-1.00) | 0.048 |  |  |  |  |  |  |
| **UNK** |  |  |  | 0.51 | (0.35-0.73) | 0.000 |  |  |  |  |  |  |
| **Income** | Not Included | | |  |  |  | Not Included | | | Not Included | | |
| **Less than $19,999** |  |  |  | 1.00 |  |  |  |  |  |  |  |  |
| **$20,000-$49,999** |  |  |  | 0.98 | (0.59-1.63) | 0.943 |  |  |  |  |  |  |
| **$50,000-$99,999** |  |  |  | 0.98 | (0.56-1.70) | 0.940 |  |  |  |  |  |  |
| **$100,000 or more** |  |  |  | 0.47 | (0.24-0.94) | 0.033 |  |  |  |  |  |  |
| **UNK/Mis** |  |  |  | 0.53 | (0.29-0.97) | 0.041 |  |  |  |  |  |  |
| **Education** | Not Included | | | Not Included | | |  |  |  |  |  |  |
| **High school or under** |  |  |  |  |  |  | 1.00 |  |  | 1.00 |  |  |
| **Some college or 2 year degree** |  |  |  |  |  |  | 0.73 | (0.49-1.08) | 0.117 | 0.80 | (0.54-1.19) | 0.272 |
| **College graduate** |  |  |  |  |  |  | 0.27 | (0.14-0.51) | <.0001 | 0.40 | (0.23-0.68) | 0.001 |
| **More than a 4 year college degree** |  |  |  |  |  |  | 0.58 | (0.33-1.03) | 0.061 | 0.56 | (0.32-0.95) | 0.032 |
| **UNK** |  |  |  |  |  |  | 0.45 | (0.12-1.73) | 0.245 | 0.45 | (0.12-1.66) | 0.230 |
| **Charlson** |  |  |  |  |  |  | Not Included | | | Not Included | | |
| **0** | 1.00 |  |  | 1.00 |  |  |  |  |  |  |  |  |
| **1** | 1.26 | (0.79-2.01) | 0.326 | 1.38 | (0.96-2.00) | 0.083 |  |  |  |  |  |  |
| **2+** | 2.45 | (1.48-4.03) | 0.001 | 2.83 | (1.79-4.47) | <.0001 |  |  |  |  |  |  |
| **UNK** | 1.69 | (0.69-4.12) | 0.249 | 1.39 | (0.64-3.03) | 0.407 |  |  |  |  |  |  |
| **Chemotherapy** |  |  |  |  |  |  |  |  |  |  |  |  |
| **No** | 1.00 |  |  | 1.00 |  |  | 1.00 |  |  | 1.00 |  |  |
| **Yes** | 1.41 | (0.93-2.13) | 0.106 | 3.25 | (2.35-4.50) | <.0001 | 1.67 | (1.17-2.37) | 0.005 | 1.72 | (1.23-2.40) | 0.002 |
| **Dx Stage** |  |  |  | Not Included | | | Not Included | | | Not Included | | |
| **Localized** | 1.00 |  |  |  |  |  |  |  |  |  |  |  |
| **Regional** | 1.94 | (1.27-2.95) | 0.002 |  |  |  |  |  |  |  |  |  |
|  |  |  |  |  |  |  |  |  |  |  |  |  |
|  | **Aching Muscles** | | | **Aching Joints** | | | **Fatigue/Lack of Energy** | | | **Hot Flashes** | | |
|  | **OR** | **95% CI** | **p-value** | **OR** | **95% CI** | **p-value** | **OR** | **95% CI** | **p-value** | **OR** | **95% CI** | **p-value** |
| **Age at diagnosis** | Not Included | | | Not Included | | | Not Included | | |  |  |  |
| **65-69** |  |  |  |  |  |  |  |  |  | 1.00 |  |  |
| **70-74** |  |  |  |  |  |  |  |  |  | 0.63 | (0.44-0.89) | 0.009 |
| **75-79** |  |  |  |  |  |  |  |  |  | 0.54 | (0.34-0.87) | 0.011 |
| **80+** |  |  |  |  |  |  |  |  |  | 0.31 | (0.17-0.56) | 0.000 |
| **Race and Ethnicity (Self-reported)** | Not Included | | | Not Included | | | Not Included | | |  |  |  |
| **White non-Hispanic** |  |  |  |  |  |  |  |  |  | 1.00 |  |  |
| **Black** |  |  |  |  |  |  |  |  |  | 2.04 | (1.13-3.69) | 0.019 |
| **Hispanic** |  |  |  |  |  |  |  |  |  | 1.43 | (0.83-2.45) | 0.197 |
| **Others** |  |  |  |  |  |  |  |  |  | 0.35 | (0.09-1.41) | 0.139 |
| **BMI** |  |  |  |  |  |  |  |  |  | Not Included | | |
| **<25** | 1.00 |  |  | 1.00 |  |  | 1.00 |  |  |  |  |  |
| **25-30** | 1.25 | (0.89-1.75) | 0.206 | 1.34 | (0.95-1.87) | 0.092 | 1.58 | (1.14-2.18) | 0.006 |  |  |  |
| **>30** | 1.62 | (1.15-2.27) | 0.006 | 2.63 | (1.88-3.69) | <.0001 | 1.60 | (1.14-2.24) | 0.007 |  |  |  |
| **Missing** | 1.55 | (0.96-2.49) | 0.070 | 1.70 | (1.06-2.72) | 0.027 | 1.35 | (0.86-2.13) | 0.196 |  |  |  |
| **Marital status** | Not Included | | |  |  |  |  |  |  | Not Included | | |
| **Married** |  |  |  | 1.00 |  |  | 1.00 |  |  |  |  |  |
| **Not married** |  |  |  | 0.99 | (0.69-1.40) | 0.941 | 0.96 | (0.67-1.35) | 0.798 |  |  |  |
| **UNK** |  |  |  | 0.67 | (0.50-0.90) | 0.007 | 0.72 | (0.54-0.95) | 0.019 |  |  |  |
| **Education** | Not Included | | | Not Included | | | Not Included | | |  |  |  |
| **High school or under** |  |  |  |  |  |  |  |  |  | 1.00 |  |  |
| **Some college or 2 year degree** |  |  |  |  |  |  |  |  |  | 0.80 | (0.55-1.15) | 0.230 |
| **College graduate** |  |  |  |  |  |  |  |  |  | 0.75 | (0.46-1.22) | 0.243 |
| **More than a 4 year college degree** |  |  |  |  |  |  |  |  |  | 0.58 | (0.36-0.93) | 0.025 |
| **UNK** |  |  |  |  |  |  |  |  |  | 3.90 | (0.77-19.70) | 0.100 |
| **Charlson** |  |  |  |  |  |  |  |  |  | Not Included | | |
| **0** | 1.00 |  |  | 1.00 |  |  | 1.00 |  |  |  |  |  |
| **1** | 1.41 | (1.03-1.92) | 0.030 | 1.48 | (1.09-2.01) | 0.013 | 1.44 | (1.06-1.95) | 0.020 |  |  |  |
| **2+** | 2.03 | (1.36-3.01) | 0.001 | 2.72 | (1.81-4.08) | <.0001 | 3.27 | (2.17-4.92) | <.0001 |  |  |  |
| **UNK** | 1.21 | (0.60-2.43) | 0.601 | 1.22 | (0.60-2.45) | 0.583 | 0.83 | (0.41-1.65) | 0.589 |  |  |  |
| **Chemotherapy** |  |  |  |  |  |  |  |  |  | Not Included | | |
| **No** | 1.00 |  |  | 1.00 |  |  | 1.00 |  |  |  |  |  |
| **Yes** | 1.61 | (1.22-2.12) | 0.001 | 1.45 | (1.10-1.92) | 0.010 | 1.59 | (1.22-2.09) | 0.001 |  |  |  |

C: Logistic Regression Models of Long-term Symptoms Reported by Breast Cancer Survivors – Symptom Interference with Daily Activities

|  | **Arm/Leg Swelling** | | | **Numbness/Tingling** | | | **Problem with Concentration** | | | **Problem with Memory** | | |
| --- | --- | --- | --- | --- | --- | --- | --- | --- | --- | --- | --- | --- |
|  | **OR** | **95% CI** | **p-value** | **OR** | **95% CI** | **p-value** | **OR** | **95% CI** | **p-value** | **OR** | **95% CI** | **p-value** |
| **Race and Ethnicity (Self-reported)** | Not Included | | |  |  |  | Not Included | | | Not Included | | |
| **White non-Hispanic** |  |  |  | 1.00 |  |  |  |  |  |  |  |  |
| **Black** |  |  |  | 1.80 | (1.04-3.13) | 0.037 |  |  |  |  |  |  |
| **Hispanic** |  |  |  | 1.14 | (0.68-1.92) | 0.612 |  |  |  |  |  |  |
| **Others** |  |  |  | 0.30 | (0.10-0.90) | 0.032 |  |  |  |  |  |  |
| **BMI** |  |  |  |  |  |  | Not Included | | | Not Included | | |
| **<25** | 1.00 |  |  | 1.00 |  |  |  |  |  |  |  |  |
| **25-30** | 1.35 | (0.78-2.34) | 0.277 | 1.39 | (0.93-2.09) | 0.111 |  |  |  |  |  |  |
| **>30** | 2.77 | (1.67-4.59) | <.0001 | 2.23 | (1.49-3.34) | 0.000 |  |  |  |  |  |  |
| **Missing** | 2.74 | (1.43-5.22) | 0.002 | 3.44 | (1.99-5.95) | <.0001 |  |  |  |  |  |  |
| **Marital status** | Not Included | | |  |  |  |  |  |  | Not Included | | |
| **Married** |  |  |  | 1.00 |  |  | 1.00 |  |  |  |  |  |
| **Not married** |  |  |  | 0.74 | (0.50-1.10) | 0.134 | 0.91 | (0.63-1.32) | 0.622 |  |  |  |
| **UNK** |  |  |  | 0.66 | (0.47-0.93) | 0.017 | 0.70 | (0.52-0.94) | 0.019 |  |  |  |
| **Income** |  |  |  | Not Included | | | Not Included | | | Not Included | | |
| **Less than $19,999** | 1.00 |  |  |  |  |  |  |  |  |  |  |  |
| **$20,000-$49,999** | 0.68 | (0.39-1.19) | 0.176 |  |  |  |  |  |  |  |  |  |
| **$50,000-$99,999** | 0.69 | (0.38-1.25) | 0.221 |  |  |  |  |  |  |  |  |  |
| **$100,000 or more** | 0.18 | (0.08-0.42) | <.0001 |  |  |  |  |  |  |  |  |  |
| **UNK/Mis** | 0.81 | (0.45-1.44) | 0.466 |  |  |  |  |  |  |  |  |  |
| **Charlson** |  |  |  |  |  |  |  |  |  |  |  |  |
| **0** | 1.00 |  |  | 1.00 |  |  | 1.00 |  |  | 1.00 |  |  |
| **1** | 1.25 | (0.80-1.96) | 0.324 | 1.55 | (1.09-2.20) | 0.015 | 1.46 | (1.06-2.01) | 0.020 | 1.27 | (0.93-1.73) | 0.130 |
| **2+** | 3.59 | (2.22-5.81) | <.0001 | 2.86 | (1.87-4.39) | <.0001 | 2.12 | (1.41-3.18) | 0.000 | 1.75 | (1.18-2.58) | 0.005 |
| **UNK** | 1.64 | (0.72-3.76) | 0.242 | 1.41 | (0.70-2.83) | 0.339 | 0.94 | (0.43-2.07) | 0.873 | 0.97 | (0.47-2.01) | 0.930 |
| **Chemotherapy** |  |  |  |  |  |  |  |  |  |  |  |  |
| **No** | 1.00 |  |  | 1.00 |  |  | 1.00 |  |  | 1.00 |  |  |
| **Yes** | 1.48 | (0.99-2.23) | 0.059 | 2.88 | (2.11-3.92) | <.0001 | 1.75 | (1.32-2.32) | 0.000 | 1.99 | (1.52-2.61) | <.0001 |
| **Dx Stage** |  |  |  | Not Included | | | Not Included | | | Not Included | | |
| **Localized** | 1.00 |  |  |  |  |  |  |  |  |  |  |  |
| **Regional** | 1.59 | (1.04-2.45) | 0.034 |  |  |  |  |  |  |  |  |  |
|  |  |  |  |  |  |  |  |  |  |  |  |  |
|  | **Aching Muscles** | | | **Aching Joints** | | | **Fatigue/Lack of Energy** | | |  |  |  |
|  | **OR** | **95% CI** | **p-value** | **OR** | **95% CI** | **p-value** | **OR** | **95% CI** | **p-value** |  |  |  |
| **BMI** |  |  |  |  |  |  |  |  |  |  |  |  |
| **<25** | 1.00 |  |  | 1.00 |  |  | 1.00 |  |  |  |  |  |
| **25-30** | 1.52 | (1.09-2.12) | 0.015 | 1.93 | (1.40-2.66) | <.0001 | 1.93 | (1.40-2.66) | <.0001 |  |  |  |
| **>30** | 1.91 | (1.36-2.70) | 0.000 | 2.58 | (1.83-3.63) | <.0001 | 2.58 | (1.83-3.63) | <.0001 |  |  |  |
| **Missing** | 1.64 | (1.03-2.60) | 0.038 | 1.82 | (1.15-2.87) | 0.011 | 1.82 | (1.15-2.87) | 0.011 |  |  |  |
| **Income** |  |  |  | Not Included | | | Not Included | | |  |  |  |
| **Less than $19,999** | 1.00 |  |  |  |  |  |  |  |  |  |  |  |
| **$20,000-$49,999** | 0.54 | (0.34-0.84) | 0.006 |  |  |  |  |  |  |  |  |  |
| **$50,000-$99,999** | 0.74 | (0.47-1.16) | 0.186 |  |  |  |  |  |  |  |  |  |
| **$100,000 or more** | 0.49 | (0.28-0.86) | 0.013 |  |  |  |  |  |  |  |  |  |
| **UNK/Mis** | 0.58 | (0.36-0.93) | 0.022 |  |  |  |  |  |  |  |  |  |
| **Charlson** |  |  |  |  |  |  |  |  |  |  |  |  |
| **0** | 1.00 |  |  | 1.00 |  |  | 1.00 |  |  |  |  |  |
| **1** | 1.54 | (1.13-2.10) | 0.007 | 1.28 | (0.94-1.73) | 0.116 | 1.28 | (0.94-1.73) | 0.116 |  |  |  |
| **2+** | 2.84 | (1.88-4.29) | <.0001 | 2.98 | (1.94-4.59) | <.0001 | 2.98 | (1.94-4.59) | <.0001 |  |  |  |
| **UNK** | 1.52 | (0.76-3.02) | 0.234 | 0.85 | (0.43-1.67) | 0.629 | 0.85 | (0.43-1.67) | 0.629 |  |  |  |
| **Chemotherapy** |  |  |  |  |  |  |  |  |  |  |  |  |
| **No** | 1.00 |  |  | 1.00 |  |  | 1.00 |  |  |  |  |  |
| **Yes** | 1.80 | (1.36-2.37) | <.0001 | 1.67 | (1.27-2.21) | 0.000 | 1.67 | (1.27-2.21) | 0.000 |  |  |  |
